# Brazilian public policy for chronic kidney disease prevention: challenges and perspectives

**DOI:** 10.11606/s1518-8787.2020054001708

**Published:** 2020-08-18

**Authors:** Patrícia Aparecida Barbosa Silva, Líliam Barbosa Silva, Joseph Fabiano Guimarães Santos, Sônia Maria Soares

**Affiliations:** I Universidade Federal de Minas Gerais Escola de Enfermagem Pós-Graduação em Enfermagem Belo HorizonteMG Brasil Universidade Federal de Minas Gerais. Escola de Enfermagem. Pós-Graduação em Enfermagem. Belo Horizonte, MG, Brasil; II Hospital Governador Israel Pinheiro Belo HorizonteMG Brasil Hospital Governador Israel Pinheiro. Belo Horizonte, MG, Brasil; III Universidade Federal de Minas Gerais Escola de Enfermagem Departamento de Enfermagem Básica Belo HorizonteMG Brasil Universidade Federal de Minas Gerais. Escola de Enfermagem. Departamento de Enfermagem Básica. Belo Horizonte, MG, Brasil

**Keywords:** Renal Insufficiency, Chronic, prevention & control, Risk Groups, Aging, Health Programs and Plans, Primary Health Care

## Abstract

Chronic kidney disease is a pathology with exponential increasing prevalence worldwide. This trend derives mainly from population aging and the growth of chronic conditions, making prevention a priority in public health. Thus, this observation instigates debates on the advances and challenges in public policies aimed at facing the progression of this disease and its risk factors in a contemporary reality that requires changes in the management models of chronic conditions. Brazilian and international experiences show that actions to prevent chronic kidney disease in risk groups remain incipient, especially in low-income countries. This area requires investment, supporting planning individualized, interdisciplinary and shared care with primary health care, as well as the user’s responsibility for their care, with proactivity and establishment and monitoring of goals to achieve satisfactory results.

## INTRODUCTION

The growing population aging and the increase in traditional risk factors, such as hypertension, diabetes and cardiovascular diseases, place chronic kidney disease (CKD) as one of the greatest challenges to public health in this century^1^. Estimates indicate overall prevalence of CKD (stages 1 to 5) in 14.3% in the general population and 36.1% in risk groups^2^. In Brazil, the estimated prevalence of CKD (stages 3 to 5) in adults is 6.7%, tripling in individuals aged 60 years or older^3^.

In 2017, CKD was responsible for 1.2 million deaths, assuming the 12th position in causes of death in the world. In the Brazilian context, this chronic condition accounts for 35,000 deaths, occupying the 10th position^4^. It is also estimated that 2.3 million to 7.1 million individuals died prematurely due to lack of access to renal replacement therapy (RRT), with higher death rates in low- and middle-income countries (in Asia, Africa and Latin America)^5^.

Thus, global efforts to increase awareness of CKD and its risk factors have mobilized several countries to tackle this chronic condition and its complications, demanding that governments design public policies that can support early identification and treatment programs, with emphasis on preventive measures that slow the progression of renal function loss^6^. The implementation of a public policy for preventing kidney diseases in the world is recent, dating from 2002, when the National Kidney Foundation published the first guideline for CKD diagnosis and treatment, in its document Kidney Disease Outcomes Quality Initiative (K/DOQI)^7^. This guideline represented an important advance in the field of nephrology, as it standardized the CKD classification system in different parts of the continents. It was also the first step towards promoting CKD awareness among care providers and health agencies, placing it as a global public health issue^8^.

However, such initiatives have shown themselves incipient in their effective implementation in risk groups, as evidenced by the multinational Global Kidney Health Atlas project, which involved 118 countries grouped among the World Bank’s four income groups (17 low-income, 33 middle-low income, 30 middle-high income and 38 high-income). This study identified failures in renal care, especially in primary care. In low-income countries, particularly in the African continent, only one third had access to serum creatinine levels and none were able to measure albuminuria and report the estimated glomerular filtration rate (eGFR), clinical parameters essential for CKD diagnosis and staging. This reality remained in high-income countries, with only 58% and 68% providing albuminuria and eGFR in primary care, respectively^9^. These results highlight the major challenge currently imposed in implementing CKD control and prevention strategies, which mainly consists in the quality and effectiveness of existing programs in primary care, as well as in the health professionals’ degree of motivation, training and permanent education, in addition to the public awareness status^10^.

Particularly in Brazil, overcoming the fragmentation and incompleteness of clinical practices and health promotion is one of the most urgent challenges to improve primary care quality in the country^11^. These weaknesses contribute in keeping the care for people with CKD focused on high complexity, as well as to the technical operational criteria of dialysis services, to the detriment of the integrality of care and health promotion, a situation that may be aggravated by the recent national health privatization and commodification projects^12^. Thus, this observation instigates the debate on the advances and challenges in Brazilian public policies aimed at facing the progression of CKD and its risk factors in a contemporary scenario that requires changes in the management models of chronic conditions.

## Brief History and Advances in Brazilian Public Policies Aimed at Facing the Progression of CKD and Its Risk Factors

In Brazil, the implementation of a public policy for preventing kidney diseases is recent, being instituted by ordinance GM/MS no. 1,168/2004^13^, which has as one of its objectives organizing a line of comprehensive care integrated in the management of the main causes for kidney injury in the Unified Health System (SUS). The recognition of health promotion and CKD prevention actions at all care levels reflects the importance of this regulation, quite different from previous policies, which dealt with the problem of CKD in a fragmented and punctual manner, prioritizing high complexity through RRT, especially in the dialysis modality by the private network^12^.

Subsequently, the Ministry of Health launched in 2006 guidelines for the Clinical Prevention of Cardiovascular, Cerebrovascular and Chronic Kidney Disease, which recommended performing early screening in primary care in risk groups, namely diabetes mellitus, hypertension and family history of CKD^14^. It was the first publication within the thematic agenda of the Ministry of Health Primary Care Journals to deal with CKD, systematically synthesizing the current knowledge on the prevention of kidney diseases (CKD staging by Cockcroft-Gault equation). Previously, these recommendations were irregular, scattered in manuals and protocols related to diabetes and hypertension. In this sense, these guidelines advanced with regards to public health policies in Brazil, as they began structuring a line of care for chronic renal patients, based on comprehensive care and greater emphasis on the primary level of health care as a gateway to SUS. Including in their scope the attributions and competencies of the health team and the referral criteria for reference and counter-reference.

Furthermore, reinforcing that the main action in preventing CKD cases is reducing and treating the main risk factors for the development of kidney injury, in 2011 the Strategic Actions Plan for Combating Chronic Noncommunicable Diseases (CNCD) was developed by the Brazilian federal government 2011–2022. Among the proposed goals, we highlight those influencing the development of kidney injury, such as reducing the rate of premature mortality in individuals under 70 years by chronic condition (including CKD) by 2% per year, reducing the prevalence of obesity in the general population, encouraging physical activity during leisure time, increasing the consumption of fruits and vegetables, reducing average salt consumption, reducing smoking prevalence and harmful alcohol consumption^15^.

Another regulation that deserves mentioning refers to ordinance no. 389 of the Ministry of Health, of March 13, 2014, which defines in greater detail the criteria for organizing the line of care for people with CKD^16^. This ordinance reinforces the importance of primary care in optimizing the management of this disease, outlining as one of the health team’s tasks performing early diagnosis and timely treatment of CKD in accordance with the Clinical Protocols and Therapeutic Guidelines, as well as care according to the Clinical Guidelines for the Care of Patients with Chronic Kidney Disease in the Unified Health System.

Such legal device^16^ advances the diagnostic criteria of CKD by including in staging the presence of microalbuminuria in the early stages of CKD, in addition to the emission of automated eGFR by clinical analysis laboratories. It also encourages promoting health professionals’ permanent education for the prevention, diagnosis and treatment of CKD and risk factors, in line with the guidelines of the National Policy for Permanent Education in Health. Thus, we observe the existence of legal subsidies that enable changes in the work process of health teams in primary care in the country, expanding the populations’ risk diagnosis capacity. Another important advance of this ordinance^16^ refers to the establishment of quality indicators for monitoring and evaluating health facilities authorized to provide health care to people with CKD under the SUS.

In 2014, based on the new technical recommendations proposed by the Kidney Disease: Improving Global Outcomes^17^ group, the Ministry of Health launched the Clinical Guidelines for the Care of Patients with Chronic Kidney Disease in the Unified Health System^18^. The main changes in relation to the 2006 guidelines^14^ include the replacement of the Cockcroft-Gault formula by the Modification of Diet in Renal Disease (MDRD) and Chronic Kidney Disease Epidemiology Collaboration (CKD-EPI) equations to measure eGFR, the description of clinical management according to the CKD stages and the availability of flowcharts for CKD assessment.

Finally, not intending to exhaust the topic, the Box presents a summary of the legal frameworks mentioned, which point to attempts to institutionalize actions aiming to disseminate and raise awareness among decision makers, health professionals and the population regarding the repercussions of CKD on the country’s health condition, today considered the “neglected epidemic of the century^10^.”

**Box:** Brazilian strategies and legal frameworks for facing chronic kidney disease.

Year	Strategy or legal framework	Major considerations
2004	Ordinance no. 1.168, of June 15, 2004^13^	Established the National Policy for the Care of Patients with Kidney Disease. Emphasizes the need to invest in actions to promote health and prevent this disease at all levels of health care, promoting the inversion of the care model. Identifying the determinants and conditioning factors of the main pathologies that lead to CKD, as well as expanding coverage for people with diabetes and hypertension are part of the federal government’s public policy agendas.
2006	Guidelines for Clinical Prevention of Cardiovascular, Cerebrovascular and Chronic Kidney Disease^14^	Brought as recommendation the early screening of risk groups (presence of diabetes, hypertension and family history of CKD) in primary care.
2011	Strategic Action Plan to Combat Chronic Noncommunicable Diseases (CNCD) in Brazil 2011–2022^15^	Aims to promote the development and implementation of effective, integrated, sustainable and evidence-based public policies for the prevention, control and care of CNCD and their risk factors. It highlights actions to prevent diabetes and hypertension, the main causes of CKD. Its axis I focuses on surveillance, monitoring and evaluation actions; axis II, prevention and health promotion; and axis III, comprehensive care.
2014	Ordinance no. 389 of March 13, 2014^16^	Defines the criteria for organizing the line of care for people with CKD, focusing on the population’s health needs coordinated by primary care and covering all levels of care. Establishes financial incentive for funding pre-dialytic outpatient care.
2014	Clinical Guidelines for the Care of Patients with Chronic Kidney Disease in the Unified Health System^18^	Establish guidelines for the care of people with CKD in the care network for people with chronic diseases. Provides recommendations to multidisciplinary teams on the care of people at risk or diagnosed with CKD, including risk stratification, prevention strategies, diagnosis and clinical management.

CNCD: chronic noncommunicable diseases; CKD: chronic kidney disease

## Challenges for the Prevention and Control of Chronic Kidney Disease and Possible Proposals

Despite all efforts made to reduce chronic conditions in Brazil, challenges still need to be overcome to ensure improved care for people with CKD. In recent decades, the logic of health privatization predominates, with increasing proposals for management changes, which advocate expanding the private space in social policies, while the State is responsible for coordinating and financing policies whose implementation would be the responsibility of institutions of a private legal nature^12^.

In this sense, creating policies based on SUS principles does not guarantee, in itself, its implementation, certifying service and ensuring legal support for the actions performed by health professionals, not least because one must provide the necessary material bases to implement them. Thus, we must establish in the myriad of health policies a connection between State, private sector and users that unites and reinforces the greater good of all, that is, health and life with dignity^19^, with minimal illness and complications.

Based the considerations made, the proposals are: 1) implementing the management of chronic conditions at the primary level, focusing on health promotion and disease prevention from the early identification of risk groups for kidney injuries, as well as reinforcing users’ self-care and autonomy; 2) improving programmatic actions of the reno-cardiovascular guideline, with delimitation of the situational diagnosis of the coverage area and systematic evaluation of care for people at risk of kidney injury; 3) encouraging health professionals to incorporate non-clinical aspects of chronic care into practice, emphasizing a care approach that recognizes the crucial role of users in managing their own health condition; 4) performing periodic clinical audits, as well as feedback with health professionals involved in care by disseminating reports with monitored data and indicators; 5) implementing automated eGRF, estimated by CKD-EPI equation, in the district laboratories of the municipalities that not yet have it – a strategy that generates no cost to local management and may reduce underdiagnosis of CKD; 6) establishing clinical management of diagnosed CKD cases, with continuous training of health professionals – the permanent education sector of each municipal health department may be responsible for the prevention of kidney diseases and management auditing to identify possible predictors that influence underreported CKD cases; 7) encouraging integrated multidisciplinary health residency programs in the field of nephrology.
