# Supplementary material for: Brazilian public policy for chronic kidney disease prevention: challenges and perspectives
Source: Rev Saude Publica. 2020 Aug 18;54:86. doi: 10.11606/s1518-8787.2020054001708 (PMC7438031; doi:10.11606/s1518-8787.2020054001708)
Supplement: Translation

# Translation: Política pública brasileira na prevenção da doença renal crônica: desafios e perspectivas

**Authors:** Patrícia Aparecida Barbosa Silva, Líliam Barbosa Silva, Joseph Fabiano Guimarães Santos, Sônia Maria Soares

**Affiliations:** I Brasil Universidade Federal de Minas Gerais. Escola de Enfermagem. Pós-Graduação em Enfermagem. Belo Horizonte, MG, Brasil; II Brasil Hospital Governador Israel Pinheiro. Belo Horizonte, MG, Brasil; III Brasil Universidade Federal de Minas Gerais. Escola de Enfermagem. Departamento de Enfermagem Básica. Belo Horizonte, MG, Brasil

**Keywords:** Insuficiência Renal Crônica, prevenção & controle, Grupos de Risco, Envelhecimento, Planos e Programas de Saúde, Atenção Primária à Saúde

## Abstract

A doença renal crônica é uma patologia com aumento exponencial em suas prevalências no mundo. Essa tendência tem ocorrido principalmente pelo envelhecimento populacional e crescimento das condições crônicas, o que faz da prevenção um tópico prioritário em saúde pública. Destarte este comentário vem instigar o debate sobre os avanços e desafios nas políticas públicas direcionadas ao enfrentamento da progressão dessa doença e seus fatores de risco em uma realidade contemporânea que requer mudanças nos modelos de gestão das condições crônicas. As experiências brasileira e internacional evidenciam que ações de prevenção da doença renal crônica em grupos de risco ainda são incipientes, especialmente em países de baixa renda. Há necessidade de investimento nessa área, apoiando a planificação do cuidado individualizado, interdisciplinar e compartilhado com a atenção primária à saúde, assim como a responsabilização do usuário pelo seu cuidado, com proatividade e estabelecimento e monitoramento de metas para atingir resultados satisfatórios.

## INTRODUÇÃO

O crescente envelhecimento populacional e o aumento dos fatores de risco tradicionais, tais como hipertensão, diabetes e doenças cardiovasculares, projetam a doença renal crônica (DRC) como um dos maiores desafios à saúde pública mundial deste século^1^. Estimativas indicam prevalência global de DRC (estágios 1 a 5) em 14,3% na população geral e 36,1% em grupos de risco^2^. No Brasil, a prevalência estimada de DRC (estágios 3 a 5) em adultos é de 6,7%, triplicando em indivíduos com 60 anos ou mais de idade^3^.

Em 2017, DRC foi responsável por 1,2 milhões de óbitos, assumindo a 12ª posição em causas de morte no mundo. No contexto brasileiro, tal condição crônica foi responsável por 35 mil mortes, ocupando a 10ª posição^4^. Estima-se ainda que 2,3 milhões a 7,1 milhões de indivíduos evoluíram a óbito prematuro pela falta de acesso à terapia renal substitutiva (TRS), com maiores taxas de óbitos em países de baixa e média renda (na Ásia, África e América Latina)^5^.

Mediante o exposto, esforços globais para aumentar a consciência da DRC e seus fatores de risco têm mobilizado vários países para o enfrentamento dessa condição crônica e suas complicações, demandando dos governantes o delineamento de políticas públicas que possam respaldar programas de identificação e tratamento precoce, com ênfase nas medidas preventivas que retardam a velocidade de progressão da perda da função renal^6^. É importante destacar que a implantação de uma política pública na prevenção das doenças renais no mundo é recente, datada de 2002, quando foi publicada a primeira diretriz para diagnóstico e tratamento da DRC pela *National Kidney Foundation*, em seu documento *Kidney Disease Outcomes Quality Initiative* (K/DOQI)^7^. Essa diretriz representou um importante avanço na área da nefrologia, pois padronizou o sistema de classificação da DRC em diferentes partes dos continentes. Também foi o primeiro passo para a promoção da consciência da DRC entre os provedores do cuidado e as agências de saúde, colocando-a como um problema de saúde pública mundial^8^.

Entretanto, tais iniciativas se mostram incipientes em sua implementação de forma efetiva nos grupos de risco, conforme evidenciado pelo projeto multinacional *Global Kidney Health Atlas*, que envolveu 118 países agrupados entre os quatro grupos de renda do *World Bank* (17 de baixa renda, 33 de renda média-baixa, 30 de renda média-alta e 38 de alta renda). Esse estudo identificou falhas no cuidado renal, principalmente no nível da atenção primária. Em países de baixa renda, particularmente na região da África, apenas um terço tinha acesso à dosagem de creatinina sérica e nenhum era capaz de dosar albuminúria e emitir laudo da taxa de filtração glomerular estimada (TFGe), parâmetros clínicos essenciais para o diagnóstico e estadiamento da DRC. Essa realidade também foi mantida em países de renda elevada, com somente 58% e 68% disponibilizando albuminúria e TFGe na atenção primária, respectivamente^9^. Esses resultados evidenciam o grande desafio que se impõe atualmente na concretização das estratégias de controle e prevenção da DRC, a qual consiste sobretudo na qualidade e efetividade dos programas existentes na atenção primária, bem como no grau de motivação, de capacitação e de educação permanente dos profissionais de saúde, além do estado de conscientização pública^10^.

Particularmente no Brasil, superar a fragmentação e incompletude das práticas clínicas e de promoção da saúde é um dos mais urgentes desafios para melhorar a qualidade da atenção primária no país^11^. Essas fragilidades concorrem para que o atendimento à pessoa com DRC esteja ainda voltado à alta complexidade, bem como aos critérios técnicos do funcionamento dos serviços de diálise, em detrimento da integralidade do atendimento e promoção da saúde, situação que poderá ser agravada com os recentes projetos de privatização e mercantilização da saúde em âmbito nacional^12^. Nesse sentido, este comentário vem instigar o debate sobre os avanços e desafios nas políticas públicas brasileiras direcionadas ao enfrentamento da progressão da DRC e seus fatores de risco em um cenário contemporâneo que requer mudanças nos modelos de gestão das condições crônicas.

## Breve Histórico e Avanços nas Políticas Públicas Brasileiras Direcionadas ao Enfrentamento da Progressão da DRC e Seus Fatores de Risco

No Brasil, a implantação de uma política pública na prevenção de doenças renais é recente, sendo instituída pela portaria GM/MS nº 1.168/2004^13^, que tem como um de seus objetivos a organização de uma linha de cuidados integrais e integrados no manejo das principais causas para lesão renal no Sistema Único de Saúde (SUS). O reconhecimento das ações de promoção da saúde e prevenção da DRC em todos os níveis de atenção reflete a importância dessa regulamentação, bem diferente das políticas anteriores, que tratavam a problemática da DRC de forma fragmentada e pontual, priorizando a alta complexidade por meio da TRS, sobretudo na modalidade dialítica pela rede privada^12^.

Posteriormente, o Ministério da Saúde lançou em 2006 as diretrizes para Prevenção Clínica de Doença Cardiovascular, Cerebrovascular e Renal Crônica, que recomendaram a realização do rastreamento precoce na atenção primária em grupos de risco, quais sejam, diabetes mellitus, hipertensão arterial e história familiar de DRC^14^. Foi a primeira publicação dentro da agenda temática dos *Cadernos de Atenção Básica* do Ministério da Saúde a versar sobre a DRC, sintetizando de forma sistemática o conhecimento da época vigente sobre a prevenção das doenças renais (estadiamento da DRC pela equação Cockcroft-Gault). Antes, essas recomendações não eram uniformes, e se pulverizavam nos manuais e protocolos relacionados ao diabetes e à hipertensão. Nesse sentido, essas diretrizes avançaram em termos de políticas de saúde pública no Brasil, à medida que iniciaram uma estruturação da linha de cuidado do paciente renal crônico, pautada na integralidade do cuidado e maior destaque para o nível primário de atenção à saúde como porta de entrada do SUS. Elas inclusive trouxeram em seu escopo as atribuições e competências da equipe de saúde e os critérios de encaminhamentos para referência e contrarreferência.

Ademais, reforçando que a principal ação na prevenção dos casos de DRC é a redução e tratamento dos principais fatores de risco para desenvolvimento de lesão renal, em 2011 foi elaborado pelo governo federal o Plano de Ações Estratégicas para o Enfrentamento das Doenças Crônicas Não Transmissíveis (DCNT) no Brasil 2011–2022. Entre as metas propostas, destacam-se aquelas que influenciam no desenvolvimento da lesão renal, como redução da taxa de mortalidade prematura em indivíduos com idade inferior a 70 anos por condição crônica (incluindo DRC) em 2% ao ano, redução da prevalência de obesidade na população geral, incentivo à prática de atividade física no lazer, aumento do consumo de frutas e hortaliças, redução do consumo médio de sal, redução da prevalência de tabagismo e consumo nocivo de álcool^15^.

Outra normativa que merece destaque refere-se à portaria nº 389 do Ministério da Saúde, de 13 de março de 2014, que define com maior riqueza de detalhes os critérios para a organização da linha de cuidado da pessoa com DRC^16^. Essa portaria reforça a importância da atenção primária na otimização da gestão desse agravo, delineando como uma das atribuições da equipe de saúde a realização de diagnóstico precoce e tratamento oportuno da DRC em conformidade com os Protocolos Clínicos e Diretrizes Terapêuticas, bem como a atenção de acordo com as Diretrizes Clínicas para o Cuidado ao Paciente com Doença Renal Crônica no Sistema Único de Saúde.

Tal dispositivo legal^16^ avança nos critérios diagnósticos da DRC ao incluir no estadiamento a presença de microalbuminúria nos estágios iniciais da DRC, além da emissão da TFGe automatizada pelos laboratórios de análises clínicas. Também incentiva a promoção da educação permanente dos profissionais de saúde para a prevenção, diagnóstico e tratamento da DRC e dos fatores de risco, em consonância com as diretrizes da Política Nacional de Educação Permanente em Saúde. Assim, observa-se a existência de subsídios legais que possibilitam mudanças no processo de trabalho das equipes de saúde na atenção primária no país, com ampliação da capacidade de diagnóstico de risco das populações. Outro importante avanço dessa portaria^16^ refere-se ao estabelecimento de indicadores de qualidade para o monitoramento e avaliação dos estabelecimentos de saúde autorizados a prestar a atenção à saúde às pessoas com DRC no âmbito do SUS.

Em 2014, baseando-se nas novas recomendações técnicas propostas pelo grupo *Kidney Disease: Improving Global Outcomes*^17^, o Ministério da Saúde lançou as Diretrizes Clínicas para o Cuidado ao Paciente com Doença Renal Crônica no Sistema Único de Saúde^18^. Como principais mudanças em relação às diretrizes de 2006^14^, destacam-se a substituição da fórmula Cockcroft-Gault pelas equações *Modification of Diet in Renal Disease* (MDRD) e *Chronic Kidney Disease Epidemiology Collaboration* (CKD-EPI) na mensuração da TFGe, a descrição do manejo clínico segundo os estágios da DRC e a disponibilização de fluxograma para avaliação da DRC.

Por fim, não pretendendo aqui exaurir o tema, é apresentada no Quadro a sumarização dos marcos legais mencionados, os quais apontam tentativas de institucionalização de ações em prol de divulgar e conscientizar os tomadores de decisão, os profissionais de saúde e a população quanto às repercussões da DRC no quadro sanitário do país, hoje considerada a “epidemia negligenciada do século”^10^.

**Quadro:** Estratégias e marcos legais brasileiros para enfrentamento da doença renal crônica.

Ano	Estratégia ou marco legal	Principais considerações
2004	Portaria nº 1.168 de 15 de junho de 2004^13^	Instituiu a Política Nacional de Atenção ao Portador de Doença Renal. Enfatiza a necessidade de investir em ações de promoção da saúde e prevenção desse agravo em todos os níveis de atenção à saúde, promovendo a inversão do modelo de assistência. Identificar os determinantes e condicionantes das principais patologias que levam à DRC, bem como ampliar a cobertura às pessoas com diabetes e hipertensão fazem parte das agendas de políticas públicas do governo federal.
2006	Diretrizes para Prevenção Clínica de Doença Cardiovascular, Cerebrovascular e Renal Crônica^14^	Trouxeram como recomendação o rastreamento precoce de grupos de risco (presença de diabetes, hipertensão e história familiar de DRC) na atenção básica.
2011	Plano de Ações Estratégicas para o Enfrentamento das Doenças Crônicas não Transmissíveis (DCNT) no Brasil 2011–2022^15^	Visa promover o desenvolvimento e a implementação de políticas públicas efetivas, integradas, sustentáveis e baseadas em evidências para a prevenção, o controle e o cuidado das DCNT e seus fatores de risco. Destaca ações de prevenção do diabetes e da hipertensão, principais causas de DRC. Em seu eixo I, enfoca ações de vigilância, monitoramento e avaliação; no eixo II, prevenção e promoção da saúde; e no eixo III, o cuidado integral.
2014	Portaria nº 389 de 13 de março de 2014^16^	Define os critérios para a organização da linha de cuidado da pessoa com DRC, com foco nas necessidades de saúde da população coordenado pela atenção básica e contemplando todos os níveis de atenção. Institui incentivo financeiro de custeio destinado ao cuidado ambulatorial pré-dialítico.
2014	Diretrizes Clínicas para o Cuidado ao Paciente com Doença Renal Crônica no Sistema Único de Saúde^18^	Estabelecem diretrizes para o cuidado às pessoas com DRC na rede de atenção às pessoas com doenças crônicas. Traz recomendações às equipes multiprofissionais sobre o cuidado da pessoa sob risco ou com diagnóstico de DRC, abrangendo estratificação de risco, estratégias de prevenção, diagnóstico e manejo clínico.

DCNT: doenças crônicas não transmissíveis; DRC: doença renal crônica

## Desafios para a Prevenção e Controle da Doença Renal Crônica e Possíveis Propostas

Apesar de todos os esforços empreendidos em direção à redução das condições crônicas no Brasil, observa-se que desafios ainda precisam ser superados para garantir a melhoria do cuidado às pessoas com DRC. Lembra-se que, nas últimas décadas, predomina a lógica privatista da saúde, havendo uma intensificação nas propostas de mudanças de gestão, que defendem a ampliação do espaço privado nas políticas sociais, cabendo ao Estado coordenar e financiar políticas cuja execução competiria às instituições de natureza jurídica privada^12^.

Nesse sentido, a elaboração de políticas com base nos princípios do SUS não garante, por si só, sua implementação, autenticando o serviço e garantindo respaldo legal para as ações desempenhadas pelos profissionais de saúde, até mesmo porque é necessário prover as bases materiais para implementá-las. Nessa perspectiva, é importante estabelecer na miríade das políticas de saúde uma conexão entre o Estado, o setor privado e os usuários que una e reforce o bem maior de todos, quais sejam, a saúde e a vida com dignidade^19^, com o mínimo de doenças e complicações.

Diante das considerações feitas, tem-se como propostas: 1) implementação do gerenciamento das condições crônicas no nível primário, com foco na promoção da saúde e prevenção de agravos a partir da identificação precoce de grupos de risco para lesões renais, assim como o fortalecimento do autocuidado e autonomia dos usuários; 2) aperfeiçoamento das ações programáticas da linha-guia renocardiovascular, com a delimitação do diagnóstico situacional da área de abrangência e a avaliação sistemática do cuidado às pessoas em risco de lesão renal; 3) incentivo dos profissionais de saúde à incorporação na prática de aspectos não clínicos do cuidado crônico, enfatizando a abordagem de cuidados que reconheça o papel crucial dos usuários na gestão de sua própria condição de saúde; 4) realização de auditorias clínicas periódicas, assim como o *feedback* com os profissionais de saúde envolvidos no cuidado a partir da divulgação de informes com os dados e indicadores monitorados; 5) implantação da TFGe automatizada, estimada pela equação CKD-EPI, nos laboratórios distritais das prefeituras que ainda não o têm instituído na localidade – uma estratégia que não gera custo à gestão local e poderá reduzir os subdiagnósticos de DRC; 6) instituição da gestão clínica dos casos diagnosticados de DRC, com treinamento contínuo dos profissionais de saúde – o setor de educação permanente de cada secretaria municipal de saúde poderá ficar responsável pela prevenção das doenças renais e auditoria de gestão para identificação de possíveis preditores que influenciam nos casos de DRC subnotificada; 7) incentivo de programas de residência multiprofissional integrada em saúde na área de nefrologia.
